# Surgical Treatment of Calcified Thoracic Herniated Disc Disease via the Transthoracic Approach with the Use of Intraoperative Computed Tomography (iCT) and Microscope-Based Augmented Reality (AR)

**DOI:** 10.3390/medicina60060887

**Published:** 2024-05-28

**Authors:** Mirza Pojskić, Miriam H. A. Bopp, Christopher Nimsky, Benjamin Saß

**Affiliations:** 1 Department of Neurosurgery, University of Marburg, 35039 Marburg, Germany; bauermi@med.uni-marburg.de (M.H.A.B.); sassb@med.uni-marburg.de (B.S.); 2 Marburg Center for Mind, Brain and Behavior (MCMBB), 35032 Marburg, Germany; nimsky@med.uni-marburg.de

**Keywords:** microscope-based augmented reality, intraoperative computed tomography, calcified thoracic herniated disc, lateral approach to the spine

## Abstract

*Background and Objectives*: The aim of this study is to present our experience in the surgical treatment of calcified thoracic herniated disc disease via a transthoracic approach in the lateral position with the use of intraoperative computed tomography (iCT) and augmented reality (AR). *Materials and Methods:* All patients who underwent surgery for calcified thoracic herniated disc via a transthoracic transpleural approach at our Department using iCT and microscope-based AR were included in the study. *Results*: Six consecutive patients (five female, median age 53.2 ± 6.4 years) with calcified herniated thoracic discs (two patients Th 10–11 level, two patients Th 7–8, one patient Th 9–10, one patient Th 11–12) were included in this case series. Indication for surgery included evidence of a calcified thoracic disc on magnet resonance imaging (MRI) and CT with spinal canal stenosis of >50% of diameter, intractable pain, and neurological deficits, as well as MRI-signs of myelopathy. Five patients had paraparesis and ataxia, and one patient had no deficit. All surgeries were performed in the lateral position via a transthoracic transpleural approach (Five from left side). CT for automatic registration was performed following the placement of the reference array, with a high registration accuracy. Microscope-based AR was used, with segmented structures of interest such as vertebral bodies, disc space, herniated disc, and dural sac. Mean operative time was 277.5 ± 156 min. The use of AR improved orientation in the operative field for identification, and tailored the resection of the herniated disc and the identification of the course of dural sac. A control-iCT scan confirmed the complete resection in five patients and incomplete resection of the herniated disc in one patient. In one patient, complications occurred, such as postoperative hematoma, and wound healing deficit occurred. Mean follow-up was 22.9 ± 16.5 months. Five patients improved following surgery, and one patient who had no deficits remained unchanged. *Conclusions*: Optimal surgical therapy in patients with calcified thoracic disc disease with compression of dural sac and myelopathy was resectioned via a transthoracic transpleural approach. The use of iCT-based registration and microscope-based AR significantly improved orientation in the operative field and facilitated safe resection of these lesions.

## 1. Introduction

Calcified hernias that fill >40% of the spinal canal are called giant thoracic disc herniations, and they usually result in myelopathy with associated neurologic symptoms. There is controversy about the surgical approach to the treatment for this pathology [1,2]. Calcified discs are usually visualized using computer tomography (CT) or CT-myelography as large osseous masses protruding into the spinal canal from the posterior edge of the adjacent thoracic vertebra. Preoperative magnetic resonance imaging (MRI) does not always show that the lesion in calcified. An anterior approach (minithoracotomy or thoracoscopic approach), i.e., the lateral approach to the spine, reduces the risk of spinal cord injury. However, a risk of damage to the lungs, pleura, and major vessels exists [1]. Large size and ossification and calcification of the thoracic discs make the treatment challenging, with further limitations being the scarce literature on the subject, which relies mostly on retrospective studies or small case series [3]. Technical challenges of the surgical treatment include the location of the disc, calcification and ossification with a restricted overview of the ventral cord, and characteristic blood supply to the thoracic cord [4]. The anterior transthoracic approach has the advantage of avoiding manipulation of the compressed spinal cord by reaching the herniated disc in front of it [5]. Approximately 4% present with myelopathy [5]. 

There is a risk of incomplete decompression and cord manipulation in the utilization of the anterolateral transthoracic approach, since it is performed from one side with limited visualization of the dura and part of the thoracic disc on the other side [6], so that intraoperative imaging such as iCT and 3D visualization of the disc with use of AR can assist in achieving optimal decompression and avoid neurological complications which can result from the incomplete resection of the disc. The utilization of the lateral approach for the resection of herniated discs for Xtreme Lateral Interbody Fusion (XLIF), as well as for corporectomy in fractures and the implantation of an expandable cage, in combination with iCT-based spinal navigation, has been previously described [7].

The aim of this study is to present our experience in the surgical treatment of calcified thoracic herniated disc disease with anterior decompression via a transthoracic transpleural approach in the lateral position with the use of intraoperative computed tomography (iCT) and augmented reality (AR) [8]. 

## 2. Materials and Methods

All patients who underwent surgery for calcified thoracic herniated disc via a transthoracic transpleural approach at our department using iCT and microscope-based AR were included in the study. Intraoperative computer tomography (iCT) has been implemented in our department since September 2016. Microscope-based AR was introduced in July 2018 as “see-through” AR, applying the head-up display (HUD) of the operating microscopes Pentero, Pentero900, and Kinevo900 (Zeiss, Oberkochen, Germany) in conjunction with the microscope element software (Brainlab, Munich, Germany), and providing an additional visualization on screens close to the surgical site using “videopass-through” AR [9]. 

**Figure 1:**
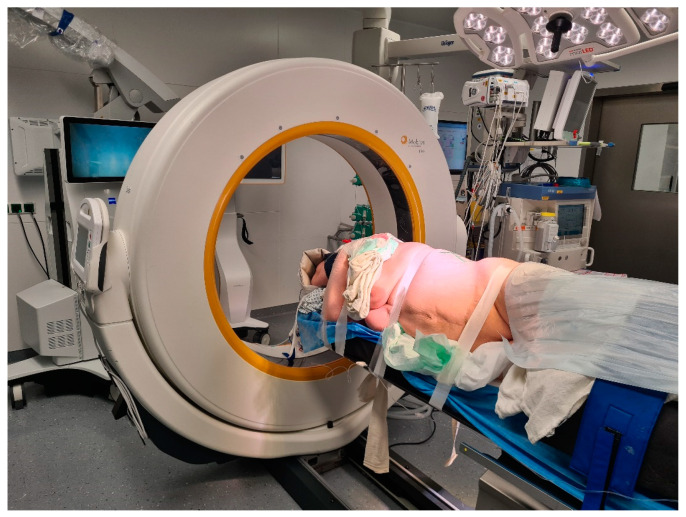
Lateral decubitus position of the patient for a left lateral transthoracic transpleural approach (patient Number 6).

**Figure 2:**
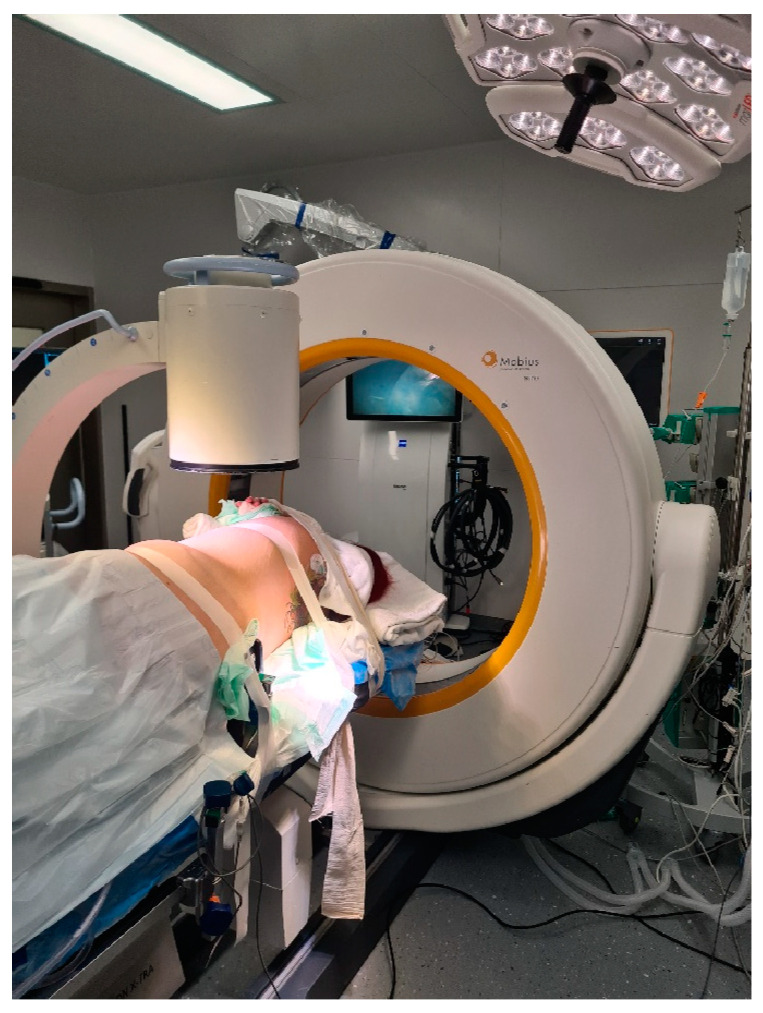
Use of standard C-arm X-ray for level definition prior to skin incision (same as Figure 1).

Operative setting and workflow have been described previously [7,10]. Briefly, our setup included: (1) approach and resection planning according to preoperative MRI and CT imaging; (2) patient positioning, neuromonitoring, and level definition; (3) iCT (registration scan); (4) fusion of preoperative and intraoperative images; (5) microsurgical AR-assisted resection; and (6) control iCT scan for extent of the resection control. Patients were positioned in the lateral decubitus position. A standard C-arm X-ray was used prior to the skin incision for level definition (Figure 1 and Figure 2). 

A standard transthoracic transpleural approach was performed. A movable 32-slice CT-scanner (AIRO, Brainlab, Munich, Germany) was used for intraoperative CT (iCT). For iCT, no patient movement was necessary. Details describing the setup were previously published, and no major modifications were necessary to apply the technique. Invasive electrophysiological monitoring, including motor evoked potentials (MEPs) and somatic sensory evoked potentials (SSEPs), was routinely used for all cases. Following definite placement of the MaXcess^®^ Retractor^®^ (^®^Nuvasive) for extreme lateral interbody fusion (XLIF), the reference array was either attached to the retractor, to the iliac crest, or proximally to the surgical field and fixed with drapes. iCT was performed after exposing the spine, without movement of the retractors. The scanner and patient were tracked during iCT to enable automatic registration. For registration scanning, dose-reduced protocols were used (helical acquisition, 33 mA) [7]. All patients received a chest tube following surgery, which was removed on the second postoperative day. 

For AR support, the HUD displays of the operating microscopes Pentero, Pentero900, and Kinevo900 (Zeiss, Oberkochen, Germany) were used. A registration array attached to the microscope allowed us to track its position. Centering the microscope above the patient reference array control of the AR calibration was performed, along with regular checks and adjustments of the AR visualization of the reference array and the optical information.

**Figure 3:**
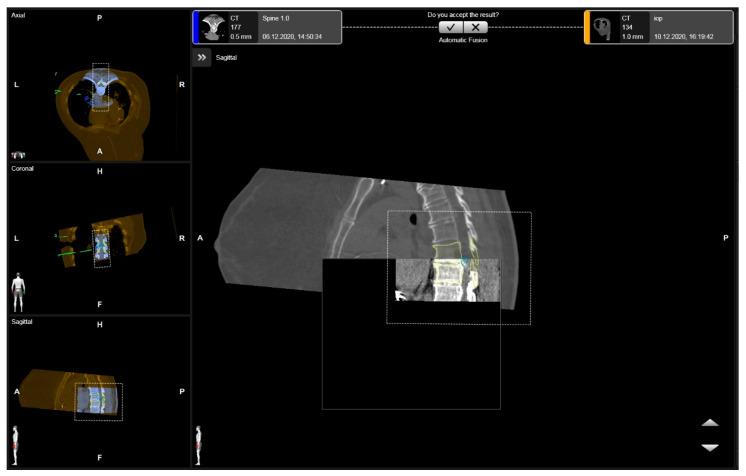
Rigid fusion of levels of interest, with the segmented vertebras in yellow and the herniated disc in blue in the axial, coronal, and sagittal views (patient number 3).

Segmentation of the herniated disc was usually manually performed in the CT and T2-weighted MRI modality (Brainlab). Vertebrae and discs were segmented automatically using CT data, with manual correction or segmentation from MRI data. The spinal cord was segmented using MRI data in a T2-weighted modality. Implants were segmented by intensity thresholding. Following iCT, elastic and rigid fusion of the preoperative and postoperative imaging was performed (Figure 3). A registration accuracy check was performed on the skin fiducials, bony landmarks, and on the retractor. A control-iCT scan was performed for control of the extent of the resection and navigation update.

## 3. Results

Six consecutive patients (five female, median age 53.2 ± 6.4 years) with calcified herniated thoracic discs (two patients on the Th 10–11 level, two patients on the Th 7–8 level, one patient on the Th 9–10 level, and one patient on the Th 11–12 level) were included in this case series. Indication for surgery included evidence of calcified thoracic discs on magnet resonance imaging (MRI) and CT with a spinal canal stenosis >40% of diameter, intractable pain, and neurological deficits, as well as MRI signs of myelopathy. Five patients had paraparesis and ataxia, and one patient had no deficit. All surgeries were performed in the lateral position via a transthoracic transpleural approach (five from the left side). Patient characteristics are summarized in Table 1.

A standard transthoracic transpleural approach was performed. A retractor was placed on the area of interest on the lateral portion of the spine. Following this, the reference array was attached at the retractor. Further modifications included the positioning of the reference array onto the iliac crest via a separate incision or the taping of the array proximally from the surgical field. iCT for automatic registration was performed, with high registration accuracy. Structures of interest, such as vertebral bodies, disc space, herniated disc, and the dural sac, were segmented, and Microscope-based AR was used. Mean operative time was 277.5 ± 156 min. The use of AR improved orientation in the operative field for identification and tailored the resection of the herniated disc and the identification of the course of the dural sac. A control-iCT scan confirmed the extent of the resection. In one patient, complications occurred, such as postoperative hematoma, and wound healing deficit occurred (Patient number 2). Mean follow-up was 22.9 ± 16.5 months. Five patients improved following surgery, and one patient who had no deficits remained unchanged.

## 4. Patients

**Figure 4:**
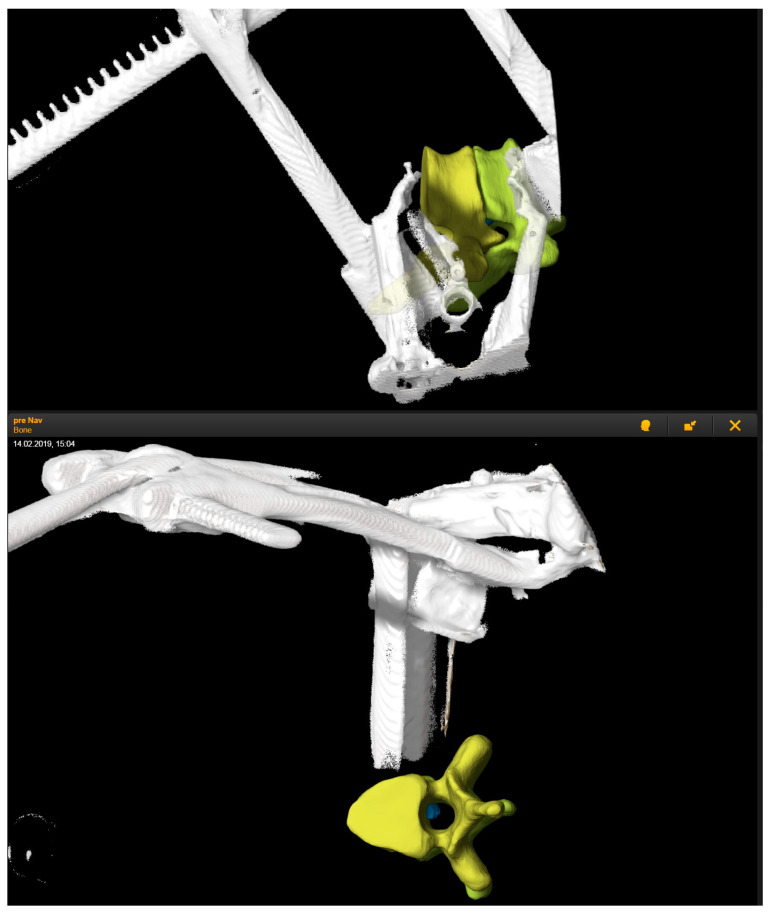
3D reconstruction of the intraoperative registration scan, which depicts the position of the retractor in relation to the segmented vertebras Th7 and Th8 in yellow and the herniated disc in blue.

Patient No. 1 received a resection of the Th7/8 disc via a left transthoracic transpleural approach. The reference array was placed at the retractor. 3D outlines of the vertebra and the segmented disc herniation were visualized in the microscope, which facilitated the resection (Figure 4).

**Figure 5:**
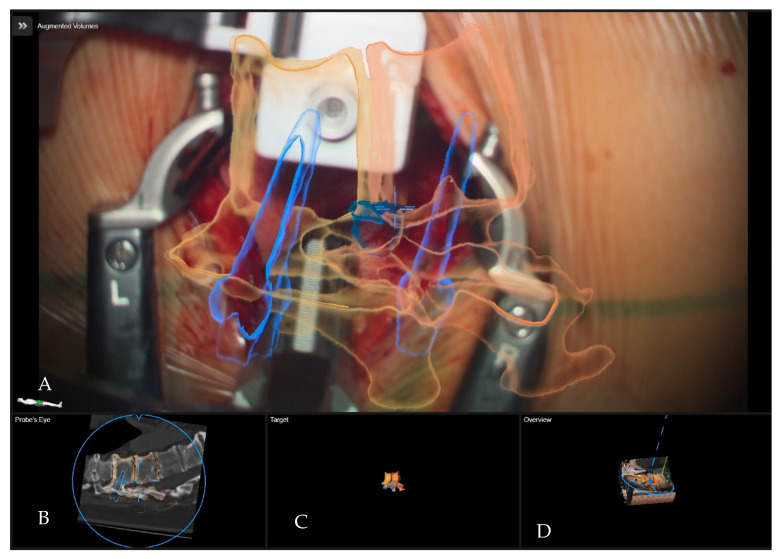
Patient number 2 with a Th11–12 thoracic disc operated via right lateral transthoracic transpleural approach following the resection of the disc. (**A**) Overview visualization depicting the position of the microscope view in relation to the segmented structures in 3D (vertebras in yellow, herniated disc in blue, screws in blue). (**B**) Probe’s-eye view with segmented structures in the iCT. (**C**) Segmented objects visualized separately in target view. (**D**) A 3D rendering of the iCT images, illustrating how the video frame is placed in relation to the image data.

Patient No. 2 underwent a two-stage surgery for a large calcified herniated disc Th11/12 with myelopathy. Dorsal stabilization of Th10–11 with decompression was performed, followed by a non-navigated resection of the herniated disc through a left transpleural approach. Follow-up CT and MRI showed incomplete resection, so a navigated resection with iCT and AR via a left transpleural approach was performed (Figure 5). In this case, the reference array was placed at the iliac crest. This patient underwent two revision surgeries: one for hematothorax on the first postoperative day, and one further revision surgery due to wound healing deficit. The patient recovered and had neurological improvement, with slight paraparesis and ataxia at follow-up. 

**Figure 6:**
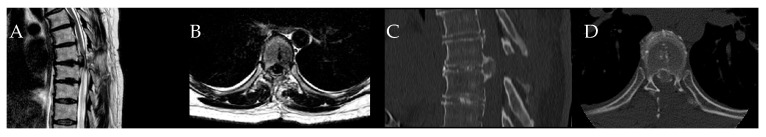
Patient No. 3. Preoperative. (**A**) T2-weighted sagittal and (**B**) T2-weighted axial MRI with preoperative (**C**) sagittal and (**D**) axial view of the calcified Th7/8 disc with myelopathy.

**Figure 7:**
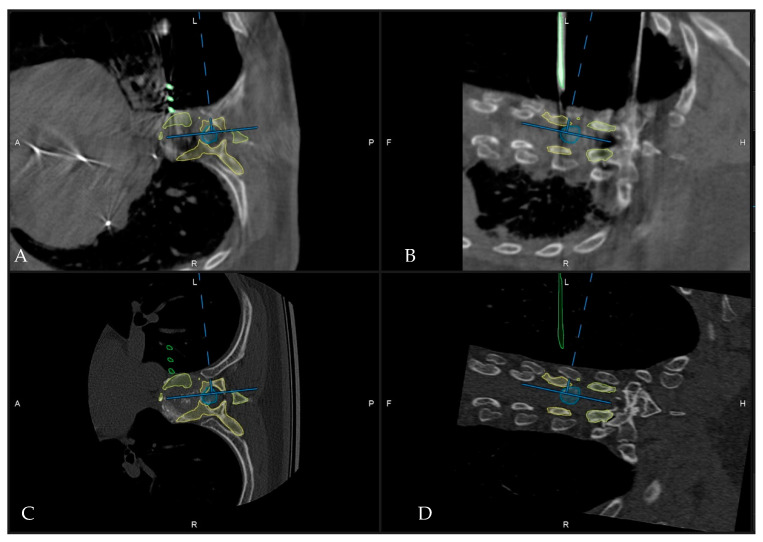
Patient No. 3, intraoperative view following the automatic registration of the patient with an iCT and the calibration of the microscope. Microscope focus is on the herniated disc (blue), with segmented vertebras Th 7/8 in yellow and the retractor in green, with a depiction in the intraoperative registration scan in the (**A**) axial and (**B**) coronal view, as well as in the preoperative scan in the (**C**) axial and (**D**) coronal view.

**Figure 8:**
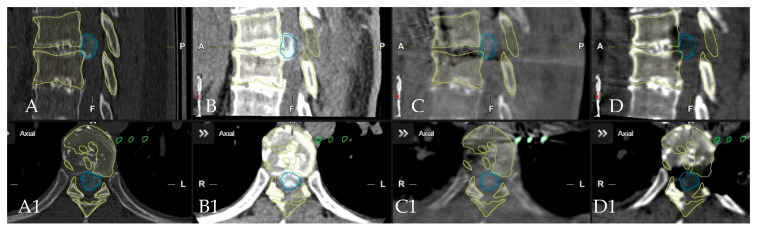
Patient No. 3, depiction of segmented vertebras in yellow and herniated disc in blue in preoperative (**A**,**B**) sagittal and (**A1**,**B1**). Axial CT with same segmented structure in intraoperative, control iCT scan following complete resection of the disc in (**C**,**D**) sagittal and (**C1**,**D1**) axial view.

Patient No. 3 underwent a left lateral transpleural approach with costotransversectomy for a large calcified herniated disc Th 7–8 with myelopathy following surgery in an external hospital, where left-sided hemilaminectomy Th7–8 was performed. Following primary surgery, the patient presented worsened paraparesis and ataxia. The reference frame was placed on the retractor arm. Complete resection of the disc was performed, and the patient improved neurologically (Figure 6, Figure 7 and Figure 8).

**Figure 9:**
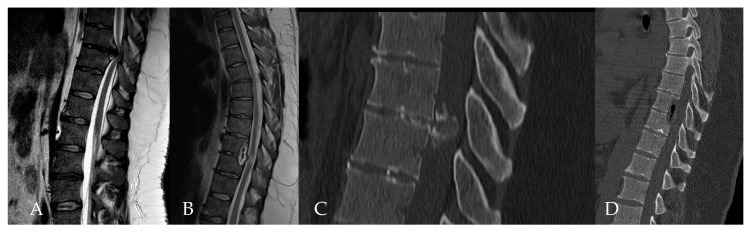
Patient No. 4 (**A**) Preoperative and (**B**) postoperative sagittal T2-weighted MRI of the thoracic spine with (**C**) preoperative and (**D**) postoperative sagittal CT of the thoracic spine following complete resection of the Th9/10 disc.

**Figure 10:**
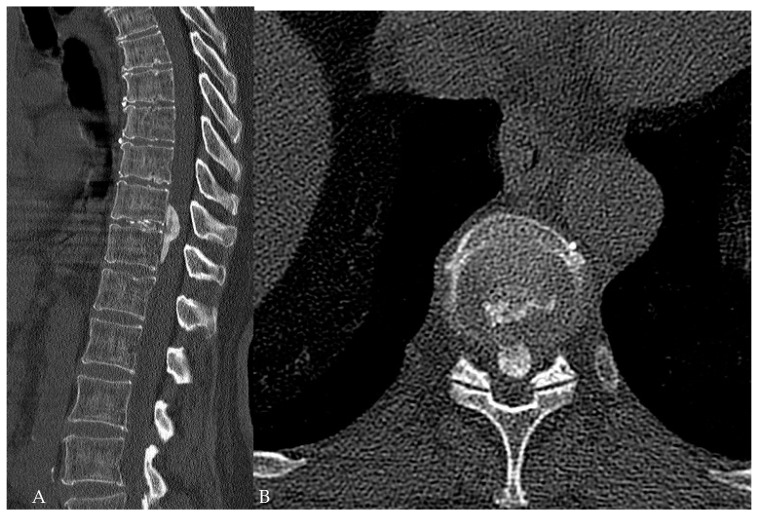
Patient No. 5, preoperative CT shows the herniated disc at the Th10–11 level with ossification of the posterior longitudinal ligament in the (**A**) sagittal and (**B**) axial view.

Patient No. 4 underwent a resection of the calcified herniated disc Th9/10 with myelopathy via a left transthoracic approach. The reference array was attached to the retractor arm. Total resection of the herniated disc was performed, and the patient recovered fully (Figure 9 and Figure 10). 

**Figure 11:**
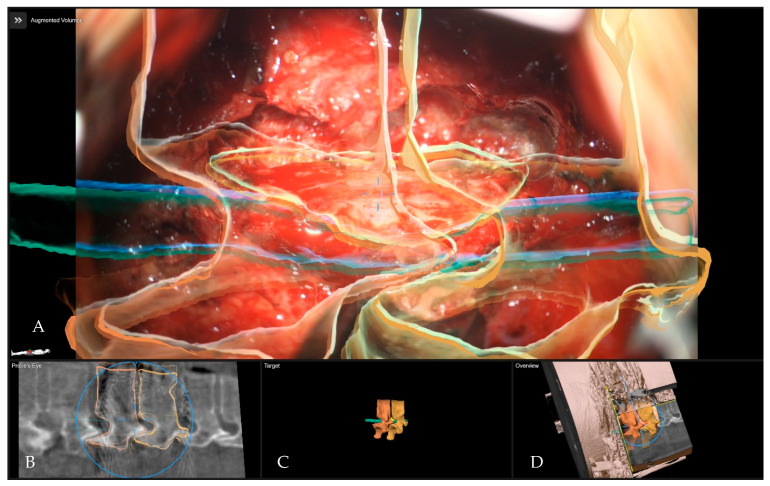
Patient No 5. with a Th10–11 thoracic disc operated via a left lateral transthoracic transpleural approach following the resection of the disc. (**A**) Overview visualization depicting the position of the microscope view in relation to the segmented structures in 3D (vertebras and disc in yellow, dural sac in green). (**B**) Probe’s-eye view, with segmented structures in the iCT. (**C**) Segmented objects visualized separately in target view. (**D**) A 3D rendering of the iCT images, illustrating how the video frame is placed in relation to the image data.

**Figure 12:**
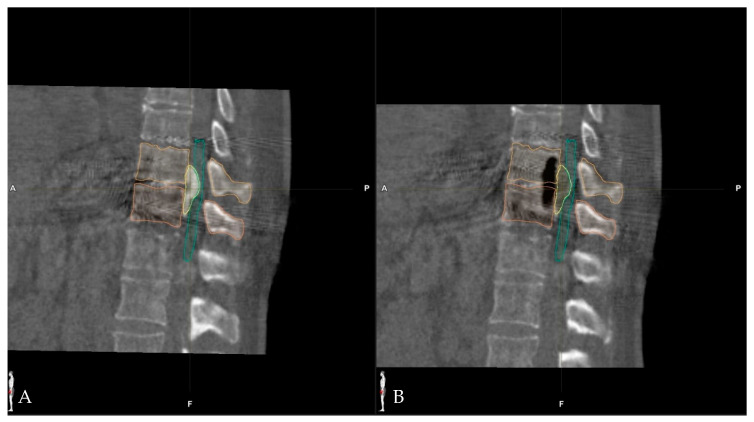
Patient No 5. sagittal view of (**A**) registration scan iCT prior to resection and (**B**) control iCT scan, which depicts the complete resection of the disc. Vertebras are segmented in orange, the disc in yellow, and the dural sac in green.

**Figure 13:**
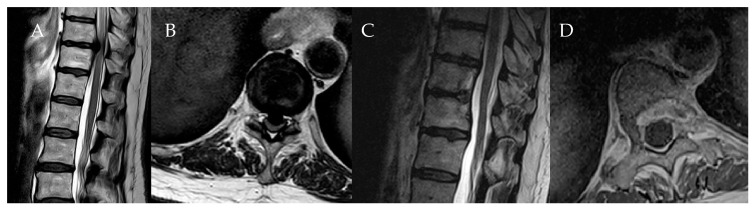
Patient No. 5. Preoperative T2-weighted (**A**) sagittal and (**B**) axial MRI with postoperative T2-weighted (**C**) sagittal and (**D**) T1-weighted post-contrast axial MRI of the thoracic spine, which shows the complete resection of the herniated disc.

Patient No. 5 is a 71-year-old female patient who experienced paraparesis, spinal ataxia, and back pain for several months. An MRI and a CT revealed a larger herniated calcified thoracic disc at the Th10–11 level. She underwent resection using a left lateral transthoracic transpleural approach. Intraoperative CT and postoperative MRI revealed the complete resection of the disc. Paraparesis was resolved, but slight spinal ataxia remained at the follow-up (Figure 10, Figure 11, Figure 12 and Figure 13, Supplemental material: Operative video). 

**Figure 14:**
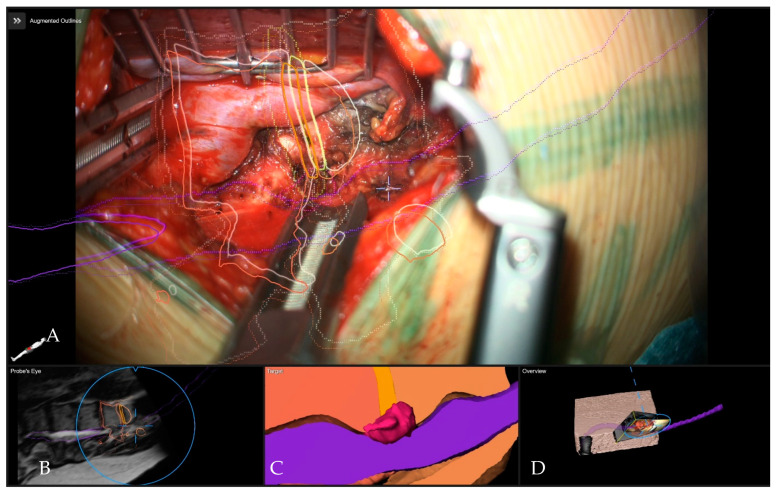
Patient No 6. with calcified T11–12 disc operated via a left lateral transthoracic transpleural approach at the beginning of microsurgical resection. (**A**) Overview visualization depicting the position of the microscope view in relation to the segmented structures in 3D (vertebras in yellow, herniated disc in red, dural sac in purple). (**B**) Probe’s-eye view with segmented structures in the MRI. (**C**) Segmented objects visualized separately in the target view. (**D**) A 3D rendering of the iCT images illustrating how the video frame is placed in relation to the image data.

**Figure 15:**
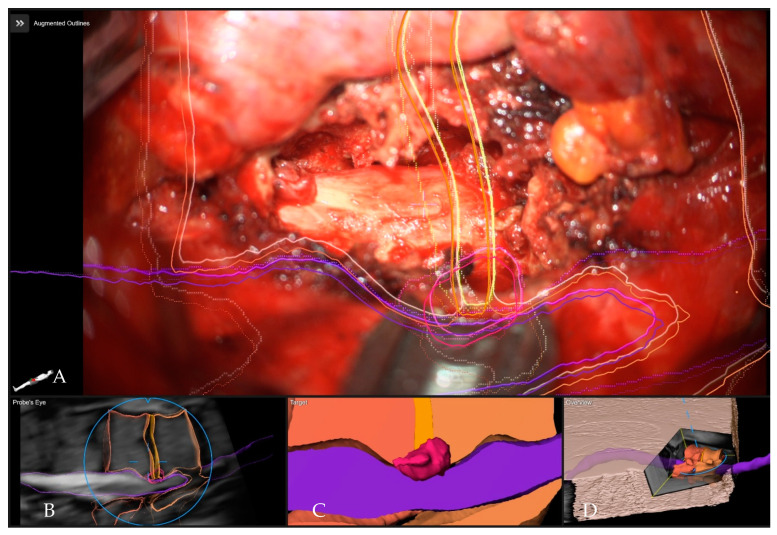
Patient No. 6 with calcified T11–12 disc operated via a left lateral transthoracic transpleural following the complete resection of the disc and anterior decompression with exposure of the dural sac. (**A**) Overview visualization depicting the position of the microscope view in relation to the segmented structures in 3D (vertebras in yellow, herniated disc in red, dural sac in purple). (**B**) Probe’s-eye view with segmented structures in the MRI. (**C**) Segmented objects visualized separately in target view. (**D**) A 3D rendering of the iCT images illustrating how the video frame is placed in relation to the image data.

**Figure 16:**
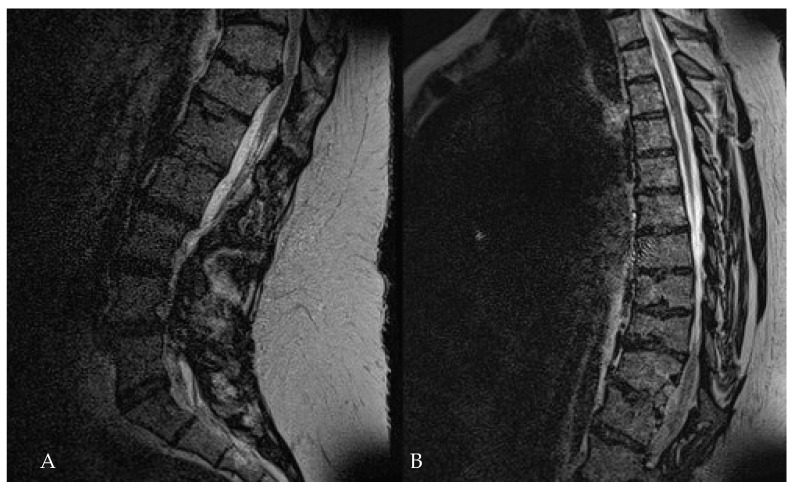
Patient No. 6. A. Preoperative T2-weighted sagittal MRI of the lumbar spine shows the herniated disc at Th11–12 level, with (**B**) the postoperative sagittal T2-weighted MRI of the thoracic spine, which shows the complete resection of the disc.

Patient No. 6 Is a 50 years old patient with calcified Th 11–12 disc with myelopathy who developed paraparesis, gait ataxia, and urinary incontinence for a period of six months. MRI revealed a large herniated disc, which was tailored-resected via a left transthoracic transpleural approach using iCT-based navigation and microscope-based AR. Intraoperative CT and postoperative MRI revealed the complete resection of the disc. Paraparesis was resolved, but slight gait ataxia remained (Figure 14, Figure 15 and Figure 16).

## 5. Discussion

### 5.1. Surgical Technique and Outcome for Resection of Calcified Thoracic Disc 

Compared to other localizations in the thoracic spine, disc herniations tend to calcify, and are more often located centrally [11]. Specific anatomical characteristics of the thoracic spine and the course of the thoracic cord and its vasculature are vital for understanding the symptoms [12]; the kyphosis of the spine makes the cord closer to the posterior side of the vertebral body so that the movement of the cord is limited. Furthermore, thoracic spinal nerve roots are tethered by the dentate ligament, which makes the cord prone to the anterior impingement [11,13]. Further factors which make the thoracic cord more vulnerable in case of a thoracic disc include a narrow spinal canal as well as the presence of the so-called “watershed zones”, which are areas of impaired blood supply [11,13]. Calcification of the nucleus pulposus in the intervertebral disc is the important radiological feature [3]. There are three types: calcium-ringed lesions, heterogeneous calcification lesions, and homogeneous calcification lesions [3]. 

There are several techniques which have been developed for the resection of the calcified thoracic discs: thoracic discectomy with ultrasound visualization via a unilateral transpedicular [14,15,16] or costotransversectomy approach [4,17]; anterior decompression and spinal fusion [3]; posterior circumspinal decompression and spinal fusion [3]; thoracotomy and hemivertebrectomy [18]; two-level corpectomy and instrumented stabilization through an open thoracotomy [2]; a mini-thoracotomy approach with anterior foraminotomy [19]; a mini-open thoracotomy and retropleural resection without the need for corpectomy or instrumentation [20]; a mini-open retropleural approach [21]; video-assisted thoracoscopic surgery [22]; thoracoscopic microdiscectomy [5,23,24]; a thoracoscopic transaxillary approach for T3–T4 herniations, with the use of cardiothoracic surgeons for access [25]; a minimally invasive dorsal approach, followed by stabilization [1]; the transthoracic endoscopic approach [26,27]; lateral retropleural discectomy [28]; transforaminal full endoscopic discectomy and foraminotomy under local anesthesia [29]; a posterior transdural approach using a three-dimensional exoscope [30]; a dual corridor method involving a tubular transthoracic/retropleural approach followed by a posterior transdural discectomy [31]; the posterior pedicle-sparing transfacet approach [14,32,33]; the full-endoscopic technique with interlaminar, extraforaminal, and transthoracic retropleural approaches [34,35]; circumspinal decompression and fusion through a posterior midline incision [6]; a minimally invasive transpedicular approach [36]; percutaneous endoscopic decompression using the T rigid bendable burr [37]; and the circumferential dural resection technique with corpectomies [38]. Large calcified discs which are located medially should be operated through an anterolateral approach, and a posterior approach can be utilized in case of soft or lateral herniations [39]. 

Otani et al. [40] and Quraishi et al. [12] were the first to describe the anterior decompression, i.e., the resection of giant calcified herniated discs via a lateral approach. The main advantage is good visualization. The best indications in soft herniated discs for thoracoscopic discectomy are lateral herniations, whereas approaches such as the oblique navigated paraspinal or posterior transdural approach can be used for central lesions [30,41]. 

Less invasive retropleural approaches (MIRA) have evolved, compared to the trans-thoracic transpleural approach, and it was reported that it has a lower complication rate such as pleural effusion and postoperative neuralgia in a series of 95 patients [42]. This approach has been described by McCormick in 1995 in a case series of 15 patients [43]. Kasliwal et al. [44] describe it for the first time for the treatment of central thoracic disc herniations. A tubular retractor system was used, and there were no approach-related complications. In some case series, such as in 33 patients by Nacar et al. [45], a mixed cohort of patients who underwent surgery with the transpleural and retropleural approach was presented. The complication rate seems to be lower with the retropleural approach. However, no consistent data in the literature exist on how many surgeries were planned to be retropleural but which ended up as transpleural due to the accidental opening of the pleura following thoracotomy due to adhesions. The use of tubular systems and, recently, endoscopy are designed to surpass open thoracotomy and lower the risk of pleural opening. Ruetten et al. described the use of full endoscopic uniportal transthoracic retropleural surgery combined with an interlaminar and extraforaminal approach in a series of 55 patients, and report no approach-related complications [46]. A combination of minimally invasive concepts, such as endoscopy or a tubular approach, with AR and navigation could improve intraoperative orientation with reduced operative time and reduced complications due to the entrance to the pleural cavity. Selective intubation with lung exclusion is one possible method for lowering the risk of pleural opening [47]. In cases without selective intubation, the mobilization of pleura can be performed from the posterior rib osteotomy site to the dorsum until the exposition of the lateral side of the thoracic spine, with a resection of the head rib for pedicle exposure [47]. Further modification is a MORP approach (mini-open retropleural approach) described by Noureldine et al. [21] in 33 patients. Although no patient required a chest tube, symptomatic pleural effusion occurred in two, and CSF leak in three, patients. In a series of six patients by Hubertus et al. [48], using the tubular system, one pneumothorax occurred as an approach-related complication. Uribe et al. [49] recommend a placement of a piece of Gelfoam over the accidental pleural defect to prevent lung protrusion without chest tube placement. Farber et al. [50] report, however, the placement of a chest tube due to a pleura violation in three out of their twelve patients who underwent thoracic disc herniation surgery via the minimaly-invasive retropleural approach. 

### 5.2. Anterolateral vs. Posterior Approach and Analysis of Clinical Outcomes

A recent study, which compared 63 patients who underwent anterior decompression to 123 patients who underwent posterior circumspinal decompression and spinal fusion for calcified thoracic discs, found a significantly shorter operation duration, a significant decrease in intraoperative blood loss, and a shorter hospital length of stay (LOS) with lower perioperative complication rate and surgery-associated complication rate in the group which underwent the posterior approach [51]. Cornips et al. [5] recommend the anterior/lateral approach for calcified herniations, since this approach enables the resection of the lesions ventrally to the cord without risk of any movement or manipulation of the cord. In their series of eight patients with acute myelopathy, all patients regained continence and ambulation. If sufficient decompression of the cord is achieved with the maintenance of the normotension during surgery, the recovery of neurological deficits is possible even if the symptoms and deficits were persistent for several days [5].

The most common symptoms are myelopathy and motor and sensory weakness [52]. Five patients in our series improved, and one patient remained unchanged. Two of our patients experienced complications, one of which was approach-related hematoma in the thoracic cavity. Barbanera et al. [53] report, in their series on seven patients, that five patients improved, one remained unchanged, and one worsened. Moran et al. [20] report no deterioration, alongside an improvement in 13/17 patients. Quint et al. describe, in their series of 167 patients who underwent a thoracoscopic resection, that after two years, roughly 80% of patients report favorable outcomes in terms of pain reduction and recovery of motor deficits, with an overall complication rate of 15.6% [24]. In a comparison of two cohorts who underwent a transthoracic and transpedicular resection, patients who underwent a mini-thoracoscopic resection had more often spasticity compared to the posterior group [39]. Roelz et al. report, in their series of 17 patients who were treated via mini thoracotomy, the successful removal of the disc in all patients, an improvement in the modified Japanese Orthopedic Association (mJOA) score from 7.9/13 to 11.1/13, and a transient post-operative neurological decline in two patients, which resolved [54]. In total, 39/43 patients who underwent a transthoracic resection for thoracic disc with myelopathy in the series of Oltulu et al. [55] experienced neurological improvement. Among the complications, there were dural tears and cerebrospinal fluid leak, as well as approach-related complications such as pneumothorax, effusion, fracture of the rib, intercostal neuralgia, and atelectasis. Pulmonary embolism and pneumonia were described as systemic complications [55].

### 5.3. Use of Navigation and Intraoperative Imaging in the Resection of Herniated Thoracic Discs

One important risk in the utilization of the anterior/lateral approach is that the side contralateral to the approach side has a limited visualization, and, therefore, incomplete decompression can occur [6]. Furthermore, fluroscopy frequently does not provide a sufficient visualization of the calcified disc, and both of these pitfalls can be avoided by the use of intraoperative imaging [53,56]. The use of iCT-guided navigation has already been described for calcified thoracic disc herniation [57]. Feigl et al. used three-dimensional planning for the complete removal of a hernia via the minimally invasive dorsal approach [1]. Similarly, Alcachupas et al. [58] used a 3D O-arm system TM for the anterolateral approach with placement of the pedicle screws. 

Nishimura et al. [32] described the use of real-time intraoperative ultrasound (RIOUS) during thoracic disc surgery in their case series of 16 patients who underwent posterior laminectomy with the pedicle-sparing transfacet approach. Four cases of patients who underwent a transpleural transthoracic resection of the herniated discs of the thoracic spine have been included in our initial report on the use of iCT-based navigation for lateral approaches to the spine [7], as well as for herniated discs and spinal canal stenosis in the lumbar spine [10]. Intraoperative ultrasound-guidance was described in a case series of 10 patients who underwent the transpedicular approach and costrotransversectomy, as well as in the posterior transpedicular approach [59]. However, the ultrasound was not navigated, and its use was limited to the visualization of the disc and control of the extent of resection [4]. Ultrasonic guidance was also used in a series of 43 patients by Saway et al. [52], although with a high percentage of incomplete resections.

Nahkla et al. [36] report a series of five patients using O-arm scans for planning and guidance of the transpedicular resection of the disc with tubular retractors. Sufficient anterior decompression and no complications were reported in all five cases. 

### 5.4. Applications of Augmented Reality for Degenerative Spine Surgery

AR use for spine surgery has been previously described [9,10,60]. Apart from publications from our research group [7,10,60], use of AR for resections of herniated discs has been described in less than 10 publications so far. Yu et al. [61] described a simulator which utilized mixed reality-based planning for percutaneous transforaminal endoscopic discectomy. Huang et al. [62] designed an augmented reality surgical navigation (ARSN) system and utilized it in percutaneous endoscopic lumbar discectomy (PELD). In a cohort of 120 patients, 10 patients underwent surgery using ARSN, with a reduction in the number of puncture attempts and number of fluoroscopies. Several case reports with the use of AR in the setting of endoscopic resections of herniated discs have been published in the previous decade [63]. 

Correct patient registration and correct microscope calibration are prerequisites for the correct visualization of the objects in the surgical field [64]. An increase in the comfort of the surgeon during the procedure, especially in re-operations, and the display of the size of the object and its relation to the surrounding structures, as well its role for educational purposes, are all described as advantages of AR [60]. Identification of the parts of the vertebra when they are not in the field of view increases confidence in the resection of the tissue, with a clear differentiation of the previously segmented herniated disc from the surrounding objects by color [65].

### 5.5. Limitations

The low number of patients and the retrospective character of the study are its drawbacks. However, this is the first study which describes the use of iCT-based navigation and microscope-based AR for tailored resections and the intraoperative extent of resection control in this disease. This case series lacks a control group which could provide an objective assessment of the use of iCT-based navigation and AR. However, all patients were treated with the best available technology, so a cohort of patients who underwent a non-navigated lateral resection or a resection using the posterior approach was not practical. Radiation-free alternatives, such as ultrasound-guidance, have not been utilized. However, their use is limited in the anterolateral approach. 

## 6. Conclusions

Optimal surgical therapy in patients with calcified thoracic disc disease with compression of the dural sac and myelopathy were resectioned via a transthoracic transpleural approach. The use of iCT-based registration and microscope-based AR significantly improved orientation in the operative field and facilitated the safe resection of these lesions. The use of iCT enabled the direct intraoperative control of the extent of the resection. 

## Figures and Tables

**Table 1:** Patient characteristics.

Patient Number	Age	Gender	Level	Approach	Preoperative Neurological Deficits	Postoperative Neurological Status	Operative Time (min)	Complications
1	63	Female	Th7–8	Left transthoracic transpleural	No deficits	Unchanged	102	None
2	50	Female	Th11–12	Right transthoracic transpleural	Paraparesis, Ataxia	Improved	213	Hematothorax, wound healing deficits
3	46	Female	Th7–8	Left transthoracic transpleural	Paraparesis, Ataxia	Improved	287	None
4	29	Male	Th9–10	Left transthoracic transpleural	Paraparesis, Ataxia	Improved	602	None
5	71	Female	Th10–11	Left transthoracic transpleural	Paraparesis, Ataxia	Improved	207	None
6	50	Female	Th11–12	Left transthoracic transpleural	Paraparesis, Ataxia	Improved	254	None

## Data Availability

A portion of the results from this manuscript were presented at https://catalog.mpil.de/vufind/EdsRecord/edselp,S2772529423007889, accessed on 24 May 2024. Abstract can be found online at: https://www.sciencedirect.com/science/article/pii/S2772529423007889, accessed on 24 May 2024; https://doi.org/10.1016/j.bas.2023.102500. (https://www.sciencedirect.com/science/article/pii/S2772529423007889, accessed on 24 May 2024). Further data are available on request to corresponding author.
